# Anticancer effects of vitamin K combined with transarterial chemoembolization in hepatocellular carcinoma, a randomized controlled trial

**DOI:** 10.1038/s41416-025-03022-4

**Published:** 2025-04-22

**Authors:** Yoshimichi Haruna, Takayuki Yakushijin, Miho Yamakawa, Tetsuo Nakazawa

**Affiliations:** 1 Department of Medical Affairs, Osaka Psychiatric Medical Center, Hirakata City, Osaka Prefecture Japan; 2 Department of Gastroenterology and Hepatology, Osaka General Medical Center, Osaka City, Osaka Prefecture Japan; 3 Liver Cancer Center, Osaka General Medical Center, Osaka City, Osaka Prefecture Japan; 4 Department of Diagnostic Imaging, Osaka General Medical Center, Osaka City, Osaka Prefecture Japan

**Keywords:** Gastrointestinal cancer, Liver cancer

## Abstract

**Background:**

We have previously reported that vitamin K dosing augments the anticancer effects of sorafenib by suppressing levels of des-γ-carboxy prothrombin, a known tumor growth and angiogenesis factor produced in HCC under sorafenib-induced ischemia. Herein, we aimed to establish whether vitamin K dosing could afford a similar anticancer effect when combined with transarterial chemoembolization (TACE).

**Methods:**

We performed a randomized controlled trial, assigning patients with unresectable HCC (1:1) to TACE + vitamin K or TACE alone groups. Co-primary endpoints were objective response rate and PFS; the secondary endpoint was safety.

**Results:**

The TACE + vitamin K group (*n* = 50) exhibited a significantly higher objective response rate than the TACE alone group (*n* = 51) (96.0% *vs*. 82.4%, *p* = 0.028). The PFS was significantly longer in the TACE + vitamin K group than that in the TACE alone group (median time: 262 days [95% confidence interval (CI), 35.8–488.2 days] *vs*. 146 days [95% CI, 111.6–180.4 days]; *p* = 0.013, hazard ratio: 0.55 [95% CI, 0.34–0.89]). There were no significant differences in the incidence of adverse events between groups.

**Conclusions:**

Compared with TACE alone, vitamin K dosing combined with TACE improved anticancer outcomes.

**Clinical trial number:**

UMIN000026404

## Introduction

Hepatocellular carcinoma (HCC) is the fourth leading cause of malignancy-related mortality worldwide, and although it is more widespread in Asia, its incidence has been rising in Western countries [1]. Depending on the cancer stage, burden, and location, various strategies have been employed to treat HCC, including resection, percutaneous ablation, transarterial chemoembolization (TACE), liver transplantation, multi-targeted tyrosine kinase inhibitors, immune checkpoint inhibitors, and anti-vascular endothelial growth factor (VEGF) or its receptor [2–4].

In a previous retrospective clinical study, we reported that combining sorafenib and vitamin K could improve anticancer outcomes [5]. In addition, the advantages of vitamin K dosing during sorafenib treatment have been confirmed in prospective studies [6]. The objective response rate was found to be significantly higher in the sorafenib + vitamin K group than that in the sorafenib alone group, and the progression-free survival (PFS) was significantly prolonged [6]. Our exploratory examination revealed that vitamin K dosing could reduce des-γ-carboxy prothrombin (DCP) levels, thereby improving anticancer outcomes [6].

DCP is not only an HCC biomarker but is also an autocrine tumor growth factor and a paracrine tumor angiogenesis factor [7, 8]. DCP binds to the cell surface receptor Met, through signaling pathways, induces the self-proliferation of HCC [7, 9]. In addition, DCP can induce the proliferation and migration of vascular endothelial cells [8]. DCP binds to the kinase insert domain receptor on vascular endothelial cells, referred to as VEGF receptor-2, resulting in tumor angiogenesis [8]. In addition, DCP plays a role in stimulating the expression of other tumor growth and tumor angiogenesis factors, including epidermal growth factor receptor, VEGF, matrix metalloproteinase-2, transforming growth factor α, and basic fibroblast growth factor [10, 11]. When HCC cells are driven to hypoxia by anticancer treatment, vitamin K transport into tumor cells is impaired [12, 13]. Accordingly, the activity of γ-glutamyl carboxylase, a vitamin K-dependent enzyme that converts DCP into prothrombin, is known to decline [12, 13]. In tumor cells, vitamin K deficiency induces the accumulation and release of DCP [12, 13]. Elevated DCP levels may facilitate the survival and growth of hypoxic tumor cells despite anticancer treatments. Conversely, pharmacological vitamin K dosing promotes the transport of vitamin K into hypoxic tumor cells and decreases DCP production [5, 6, 12]. As observed with vitamin K dosing combined with sorafenib [5, 6], the vitamin K-induced decline in DCP levels may enhance the anticancer action of TACE. Herein, we performed a prospective, randomized, controlled trial to evaluate the potential advantage of vitamin K dosing combined with TACE against unresectable HCC.

## Patients and methods

### Patients

We conducted a randomized, open-label, single-center study assessing unresectable HCC at the Osaka General Medical Center, Osaka, Japan. Patients were enrolled if they met the following inclusion criteria: ≥ 18 years of age, ≤89 years of age, a life expectancy of ˃12 weeks, maximum tumor diameter of ≤10 cm, ≤2 prior TACE sessions, Eastern Cooperative Oncology Group performance status of 0 or 1, and Child-Pugh score of A to B7. Patients were excluded if they had diffuse tumor lesions, extrahepatic metastasis, macroscopic vascular invasion, previous or concurrent malignancy, or ongoing systemic chemotherapy for HCC. In addition, patients taking warfarin, in whom vitamin K is contraindicated, were excluded.

### Randomization

Using a computer-generated randomization scheme, patients were randomly assigned in a 1:1 ratio to TACE + vitamin K or TACE alone, stratified based on meeting or not meeting Milan criteria (one nodule, diameter of ≤5 cm; or 3 nodules or less, diameter of ≤3 cm) and prior TACE (naïve *vs*. once or twice).

### Study design and blinding

This prospective, randomized, open-label, single-center study was conducted at the Liver Cancer Center of the Osaka General Medical Center. The members of the Liver Cancer Center include hepatologists from the Department of Laboratory Medicine, the Department of Gastroenterology and Hepatology, and radiologists from the Department of Diagnostic Imaging. The hepatologists screened eligible patients, provided vitamin K to patients in the TACE + vitamin K group, and performed follow-up and clinical assessments after TACE. Radiologists performed TACE and evaluated radiological images obtained by computed tomography (CT) and magnetic resonance imaging (MRI) before and after TACE. Radiologists were blinded to the vitamin K dosing to avoid inducing any bias in TACE performance or the assessment of the radiological imaging.

### Treatment protocol

Patients assigned to the TACE + vitamin K group were orally administered 45 mg of vitamin K2 (menatetrenone; GLAKAY, Eisai Co., Ltd., Tokyo, Japan) daily for 28 days, initiated on the day of TACE. Conventional TACE or drug-eluting bead TACE was performed. In cTACE, intra-arterial injection of lipiodol, epirubicin, or cisplatin was followed by arterial embolization with Gelpart. Considering drug-eluting bead TACE, epirubicin-eluting beads (DC beads; Eisai Co., Ltd., Tokyo, Japan) were injected transarterially. Anti-tumor outcomes were evaluated four weeks after TACE by dynamic CT using contrast enhancement or gadolinium-ethoxybenzyl-diethylenetriamine pentaacetic acid-MRI using modified Response Evaluation Criteria in Solid Tumors [14]. Subsequently, radiological assessments were performed every three months until progressive disease was observed.

### Study endpoints

To assess the efficacy of vitamin K combined with TACE, the primary endpoints were the objective response rate (ORR) and PFS, which were defined as the time from randomization to radiological progression or death from any cause. The patients were considered censored when TACE was repeated, or other sequential therapies were administered for viable residual tumors. The secondary endpoint was safety, assessed with National Cancer Institute Common Terminology Criteria for Adverse Events, version 5.0.

### Statistical analysis

The sample size of the present study was calculated based on a retrospective examination of TACE-treated patients administered vitamin K for osteoporosis at the Osaka General Medical Center (unpublished data). We estimated that the ORR was 0.9 and 0.6 for patients treated with TACE + vitamin K and those treated with TACE alone, respectively. Ninety-eight patients were required to detect a significant difference with 90% power and a two-sided alpha of 0.05. Therefore, we planned to enroll approximately 100 patients, 50 in the TACE + vitamin K group and 50 in the TACE alone group. PFS was assessed using the log-rank test. Survival curves and median PFS are shown using the Kaplan–Meier method. HRs and confidence intervals (CI) were estimated using a Cox proportional hazards model. The ORR, disease control rates, and incidence of adverse events were assessed using the chi-square or Fisher’s exact tests. The Mann–Whitney U test, chi-square test, or Fisher’s exact test were used to analyze baseline characteristics. The Mann–Whitney U test was used to compare the event numbers of rapid and crucial recurrence or serum DCP levels before and four weeks after TACE. Two-sided *p*-values were considered statistically significant at *p* < 0.05.

## Results

### Patients’ characteristics

**Fig. 1:**
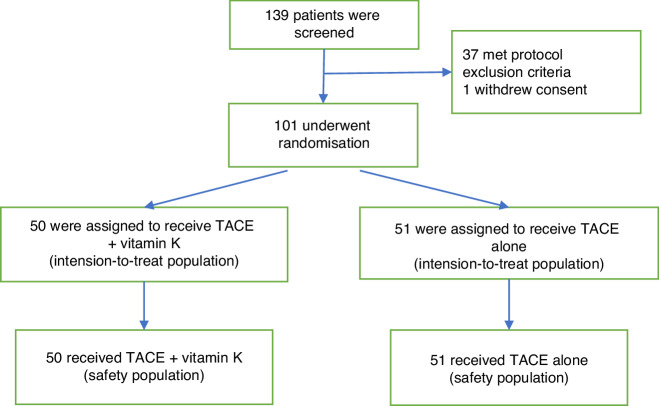
Trial profile. TACE transarterial chemoembolization.

From March 2017 to December 2021, 139 patients were screened for study enrollment. In total, 101 screened patients were enrolled and randomized (Fig. 1). There were no significant differences in demographic and clinical characteristics, including Child-Pugh scores, BCLC stages, tumor size, number of tumor nodules, macroscopic features of tumors (simple nodular type, simple nodular type with extranodular growth, and confluent multinodular type), and IVR types (conventional TACE or drug-eluting bead TACE) between the TACE + vitamin K and TACE alone groups (Table 1). Data obtained until the cutoff date (June 30, 2022) were analyzed.

**Table 1:** Baseline characteristics of patients.

Variables	TACE + vitamin K (*n* = 50)	TACE alone (*n* = 51)	*p*-value
Age, median (range), years	79.5 (61–87)	79 (53–89)	0.43
Sex, *n* (%)			0.71
Male	36 (72.0)	35 (68.6)	
Female	14 (28.0)	16 (31.4)	
ECOG performance status, *n* (%)			0.43
0	41 (82.0)	40 (78.4)	
1	9 (18.0)	11 (21.6)	
Etiology of chronic liver disease, *n* (%)			0.44
HCV infection	18 (36.0)	20 (39.2)	
HBV infection	3 (6.0)	2 (3.9)	
Alcohol	10 (20.0)	9 (17.6)	
NASH	15 (30.0)	13 (25.5)	
Other	4 (8.0)	7 (13.7)	
Child-Pugh score, *n* (%)			0.73
5	24 (48.0)	27 (52.9)	
6	14 (28.0)	15 (29.4)	
7	12 (24.0)	9 (17.7)	
AFP (ng/mL), *n* (%)			0.75
< 200	42 (84.0)	44 (86.3)	
≧200	8 (16.0)	7 (13.7)	
DCP (mAU/mL), *n* (%)			0.47
< 100	30 (60.0)	27 (52.9)	
≧100	20 (40.0)	24 (47.1)	
BCLC stage			0.83
A	28 (56.0)	29 (56.9)	
B	13 (26.0)	11 (21.6)	
C	9 (18.0)	11 (21.6)	
Milan criteria, *n* (%)			0.90
Within	31 (62.0)	31 (60.8)	
Beyond	19 (38.0)	20 (39.2)	
Up-to-7 criteria, *n* (%)			0.70
Within	37 (74.0)	36 (70.6)	
Beyond	13 (26.0)	15 (29.4)	
Macroscopic features of tumors, *n* (%)			0.07
Simple nodular	37 (74.0)	44 (86.3)	
Simple nodular with extra nodular growth	7 (14.0)	6 (11.8)	
Confluent multinodular	6 (12.0)	1 (2.0)	
Number of tumors, *n* (%)			0.28
Single	19 (38.0)	25 (49.0)	
2–9	28 (56.0)	24 (47.1)	
≧10	3 (6.0)	2 (3.9)	
Maximum diameter of tumors, *n* (%)			0.15
<50 mm	46 (92.0)	42 (82.4)	
≧50 mm	4 (8.0)	9 (17.6)	
Prior TACE session, *n* (%)			0.91
0	28 (56.0)	28 (54.9)	
1–2	22 (44.0)	23 (45.1)	
IVR			0.37
cTACE	47 (94.0)	46 (90.2)	
DEB TACE	3 (6.0)	5 (9.8)	

*AFP* α-fetoprotein, *DCP* des-γ-carboxy prothrombin, *ECOG* Eastern Cooperative Oncology Group, *HBV* hepatitis B virus, *HCV* hepatitis C virus, *NASH* nonalcoholic steatohepatitis, *IVR* interventional radiology, *cTACE* conventional transarterial chemoembolization, *DEB-TACE* drug-eluting beads transarterial chemoembolization.

### Efficacy outcomes

**Fig. 2:**
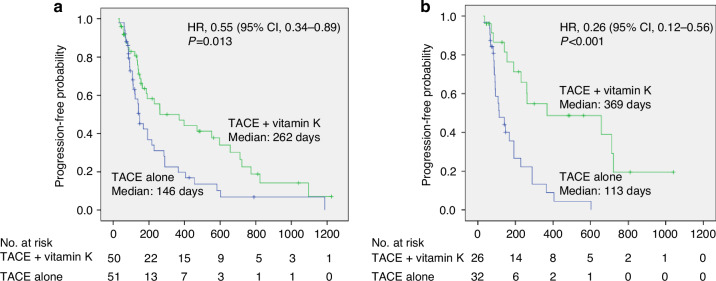
Kaplan-Meier curves of progression-free survival. **a** Kaplan-Meier estimate for all patients. **b** Kaplan-Meier estimate for  patients with baseline DCP levels $\geqq$100 mAU/mL or female patients. HR hazard ratio, DCP des-γ-carboxy prothrombin, TACE transarterial chemoembolization.

Considering the best responses in the TACE + vitamin K group, 31 (62.0%) patients achieved complete response (CR), 17 (34.0%) achieved partial response (PR), and 2 (4.0%) had stable disease (SD), with no patients exhibiting progressive disease (PD). Considering the TACE alone group, 24 (47.1%) patients achieved CR, 18 (35.3%) patients achieved PR, 5 (9.8%) achieved SD, and 4 (7.8%) presented with PD. The ORR was significantly higher in the TACE + vitamin K group than that in the TACE alone group, i.e., 48/50 (96.0%) and 42/51 (82.4%), respectively (*p* = 0.028). The disease control rate was 50/50 (100.0%) and 47/51 (92.2%) in the TACE + vitamin K and TACE alone groups, respectively (*p* = 0.061) (Supplementary Table S1). The PFS was significantly longer in the TACE + vitamin K group than that in TACE alone group (median [95% CI] days: 262 [35.8–488.2] *vs*. 146 [111.6–180.4], HR 0.55 [0.34–0.89], *p* = 0.013) (Fig. 2a).

**Fig. 3:**
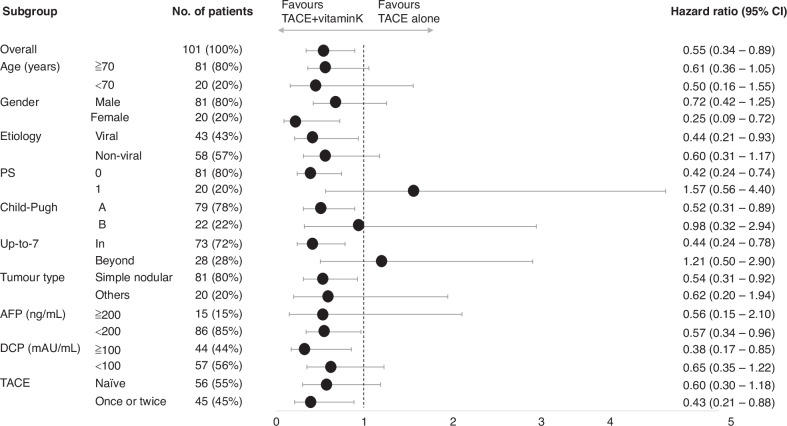
Forest plot of progression-free survival in the subgroups; TACE + vitamin K dosing group *vs*. TACE alone group. CI confidence interval, TACE transarterial chemoembolization, PS Eastern Cooperative Oncology Group performance status, AFP α-fetoprotein, DCP des-γ-carboxy prothrombin.

Based on the subgroup analysis (Fig. 3), treatment with TACE + vitamin K afforded favorable results in female patients (HR of males and females: 0.72 *vs*. 0.25) (Supplementary Fig. S1A, B), patients within up-to-7 criteria (HR of within the up-to-7 criteria and beyond: 0.44 *vs*. 1.21)(Supplementary Fig. S1C, D), and those with a better background (HR of PS-0 and PS-1: 0.42 *vs*. 1.57, HR of Child-Pugh classification A and B: 0.52 *vs*. 0.98). In contrast, elevated baseline serum DCP levels were favorable to achieve enhanced anticancer effects mediated by vitamin K dosing (HR of DCP ≧100 and <100 mAU/mL: 0.38 *vs*. 0.65) (Supplementary Fig. S1E, F). Male patients with baseline serum DCP levels ≥100 mAU/mL derived a significant benefit from Vitamin K combination therapy (HR: 0.36, *p* = 0.046), whereas those with baseline serum DCP levels <100 mAU/mL did not (HR: 1.04, *p* = 0.924). Figure 2b presents Kaplan–Meier curves of PFS in patients with baseline DCP ≧100 mAU/mL or female patients, who seem to be most favorable for TACE + vitamin K (median [95% CI] days: 369 [0–791.3] *vs*. 113 [57.5–168.5], HR 0.26 [0.12–0.57], *p* < 0.001).

We further examined differences in recurrence after TACE between the two groups, focusing on rapid and crucial recurrence, which critically impacts disease progression and prognosis. Herein, we defined rapid and crucial recurrence as the emergence of macroscopic vascular invasion (Vp4 or Vp3), multicentric intrahepatic recurrence (an increase of ˃10 nodules compared with the latest radiological image), massive infiltrative tumor growth, extrahepatic spread, or abrupt death due to rapid tumor progression, within 120 days of TACE. In the TACE + vitamin K group, 4 patients (8%) experienced 4 events of rapid and crucial recurrence, whereas 11 patients (22%) in the TACE alone group experienced 14 events of rapid and crucial recurrence. Rapid and crucial recurrence was observed more frequently in the TACE alone group than in the TACE + vitamin K group (*p* = 0.049) (Table 2).

**Table 2:** Event numbers for rapid and crucial recurrence within 120 days post-TACE.

Event of recurrence	TACE + vitamin K group	TACE alone group
Macroscopic vascular invasion^a^	0	3
Multicentric intrahepatic recurrence	3	4
Massive infiltrating recurrence	0	4
Extrahepatic spread	0	3
Death due to rapid tumor progression	1	0
Total events^b^	4	14

*TACE* transarterial chemoembolization.

^a^Portal vein tumor thrombosis: Vp4 or Vp3.

^b^Rapid and crucial recurrence was observed more frequently in the TACE alone group than that in the TACE + vitamin K group (*p* = 0.049).

Overall survival tended to be longer in the TACE + vitamin K group compared to the TACE-alone group (median: not reach *vs*. 1169 days, HR: 0.52 [0.24–1.13], *p* = 0.092). However, overall survival analysis is still immature (Supplementary Fig. S2).

### Changes in serum DCP levels before and four weeks after TACE

Given that DCP plays a role in mediating the enhanced anticancer effects of vitamin K dosing, an exploratory analysis was performed to determine changes in serum DCP levels before and four weeks after TACE (Supplementary Fig. S3). No significant difference was observed in baseline serum DCP levels between the TACE + vitamin K and TACE alone groups (*p* = 0.42) (median [range] mAU/mL, 58.1 [17.7–28093.0] *vs*. 83.2 [19.0–38999.2]). Four weeks after TACE, all patients in the TACE + vitamin K group exhibited markedly decreased serum DCP levels, which were below the normal upper limit (<40 mAU/mL). We noted a significant difference between the TACE + vitamin K group and TACE alone group (*p* < 0.001) (median [range] mAU/mL, 18.1 [8.1–36.4] *vs*. 28.9 [9.0–3170.7]).

### Safety outcomes

Table 3 shows the incidence of adverse events observed four weeks after TACE, which occurred in at least one patient in either group. Adverse events were assessed using the National Cancer Institute Common Terminology Criteria for Adverse Events, version 5.0. No relevant difference in the incidence of adverse events was observed between the TACE + vitamin K group and TACE alone group. Grade 3 or 4 adverse events were noted in 56.0 and 58.8% of patients in the TACE + vitamin K and TACE alone groups, respectively. No adverse events specific to vitamin K were observed.

**Table 3:** Incidence (percent) of adverse events observed within four weeks after TACE^a^.

Adverse event	TACE + vitamin K		TACE alone		*p*-value	
	Any grade	Grade 3/4	Any grade	Grade 3/4	Any grade	Grade 3/4
Overall incidence	100.0	56.0	100.0	58.8	NA	0.77
AST elevation	90.0	42.0	86.2	43.1	0.56	0.91
ALT elevation	94.0	24.0	82.4	31.4	0.07	0.41
Bilirubin elevation	42.0	0	41.2	0	0.93	NA
Platelet decrease	74.0	16.0	64.7	7.8	0.31	0.21
Anemia	88.0	4.0	84.3	3.9	0.59	0.68
Neutropenia	12.0	4.0	5.9	2.0	0.23	0.49
Fever	50.0	2.0	43.1	0	0.49	0.50
Nausea	2.0	0	0	0	0.50	NA
Appetite loss	66.0	16.0	64.7	13.7	0.89	0.75
Gastrointestinal bleeding	2.0	2.0	2.0	0	0.75	0.50
Creatinine increase	26.0	0	27.5	0	0.87	NA
Abdominal pain	38.0	0	45.1	0	0.47	NA

*ALT* alanine transaminase, *AST* aspartate aminotransferase, *NA* not applicable, *TACE* transarterial chemoembolization.

^a^Adverse events that occurred in at least one patient in either group are listed. The patients were assessed using the Common Terminology Criteria for Adverse Events, version 5.0.

## Discussion

The present study was a prospective, randomized, open-label, single-center trial. To the best of our knowledge, this is the first study to establish the potential anticancer advantages of vitamin K dosing in combination with TACE. This study was planned and performed based on the hypothesis that suppressing DCP levels using vitamin K would facilitate the TACE-induced hypoxic damage in HCC. In surviving HCC cells, hypoxia induces the production of DCP [13], an angiogenic and tumor growth factor [7–11]. Therefore, reduced DCP production owing to vitamin K administration may decrease the incidence of recurrence after TACE.

In Japan, patients with cirrhosis frequently receive vitamin K for concurrent osteoporosis, given that vitamin K can sustain bone mineral density [15]. Commercially available oral vitamin K2 capsules can be safely administered at 45 mg daily to prevent osteoporotic fracture. Therefore, we selected the 45 mg daily dose in the present study. Given that tumor neovessels can be rapidly reformed three weeks after transarterial embolization [16], we administered vitamin K from day 1 to day 28 after TACE. As the study was performed at a single center and the sample size was small, we carefully planned the trial, ensuring randomization and blinding. Tumor size/number and numbers of prior TACE were estimated as the most influential factors on TACE outcomes [17]. Thus, patients were randomized 1:1 to the TACE + vitamin K group or the TACE alone group, stratified using the Milan criteria and the number of prior TACE procedures. Radiologists at the Department of Diagnostic Imaging, Osaka General Medical Center, performed TACE, subsequently assessing the anticancer effects mediated by TACE using radiological evaluations. The radiologists were blinded to the administration of vitamin K to avoid possible bias.

Regarding co-primary endpoints, the ORR of the TACE + vitamin K group was significantly higher than that of the TACE alone group (96.0 *vs*. 82.4; *p* = 0.028), and PFS was significantly longer in patients treated with vitamin K combined with TACE than in those treated with TACE alone (median: 262 days *vs*. 146 days; HR = 0.55, *p* = 0.013). Furthermore, we confirmed the secondary endpoint, i.e., the safety of vitamin K dosing. There were no significant differences in adverse events between the TACE + vitamin K and TACE alone groups.

Subgroup analysis revealed significant factors related to favorable outcomes with combined vitamin K dosing and TACE. Vitamin K dosing was advantageous in patients within up-to-7 criteria but not in those beyond up-to-7 criteria. Notably, TACE is considered most suitable in the intermediate stage for up-to-7 criteria; hence, most patients undergoing TACE would benefit from vitamin K dosing. The exploratory analysis further revealed that the vitamin K-mediated prolongation of PFS was associated with baseline serum DCP levels. Vitamin K administration could afford more favorable outcomes in patients with high baseline serum DCP levels (≧100 mAu/mL) than in those with low baseline serum DCP levels (<100 mAU/mL). This finding may be attributed to DCP playing a predominant role in driving the progression of HCC in patients with high DCP levels. Therefore, the robust suppression of DCP production by vitamin K dosing may exert enhanced anticancer effects in the patients. Interestingly, we observed a difference in the anticancer effects mediated by vitamin K dosing in males and females. Vitamin K dosing induced more favorable outcomes in females than those in males (HR: 0.25 *vs*. 0.72). Vitamin K tends to exert a greater biological action in females than that in males, consistent with the anti-osteoporosis or anti-heart failure effects observed in females but not in males [18, 19]. The estrogen is thought to help prevent the occurrence and development of HCC [20]. Additionally, vitamin K was shown to enhance estrogen function [21]. Therefore, vitamin K may compensate for the declining estrogen levels in post-menopausal women, helping to suppress tumor growth.

Considering these factors together, among patients (with baseline serum DCP levels ≧100 mAU/mL or female), there is a remarkable difference in PFS between the TACE + vitamin K group and TACE alone group (median:369 days *vs*. 113days; HR:0.26, *p* < 001).

The TACE + vitamin K and TACE alone groups exhibited reduced serum DCP levels, reflecting tumor regression after TACE. However, the TACE + vitamin K group showed substantially lower DCP levels than the TACE alone group. All patients in the TACE + vitamin K group showed serum DCP levels less than the normal upper limit, despite including PR and SD. This finding confirmed the robust vitamin K-mediated suppression of DCP.

Murata et al. have reported that hypoxia transforms HCC cells into ones with epithelial-to-fibroblastoid conversion or epithelial-mesenchymal transition, which increases DCP production [13]. The elevated DCP levels in the intratumor microenvironment after TACE may promote proliferation of the transformed tumor cells and lead to intrahepatic and extrahepatic HCC progression. We subsequently focused on the incidence of rapid and crucial recurrence, including massive vascular invasion, multicentric intrahepatic recurrence, massive tumor infiltration, and extrahepatic spread, within 120 days of TACE. We found that vitamin K administration decreased the frequency of such drastic recurrence after TACE.

TACE-induced hypoxia can induce various angiogenic factors, and the induction of tumor angiogenesis could eradicate the anticancer effect of TACE [22–24]. Several clinical trials have been conducted to clarify the possible advantages of molecular-targeted agents, including sorafenib or lenvatinib, combined with TACE, based on the hypothesis that molecular-targeted agents may suppress tumor angiogenesis after TACE and enhance anticancer effects [25–33]. Although some trials have demonstrated the efficacy of molecular-targeted agent combinations, patients experienced adverse events related to these agents. In contrast, vitamin K dosing did not result in additional adverse events and was cost-effective, as vitamin K is substantially cheaper when compared with molecular-targeted agents. Combining various multi-tyrosine kinase inhibitors, immune checkpoint inhibitors, or anti-VEGF antibodies with TACE may afford beneficial outcomes. In addition to the benefits of such combination treatments, vitamin K dosing could exert a further enhanced anticancer effect. According to the manufacturers’ drug information, Vitamin K is not listed as a contraindication for co-administration with multi-tyrosine kinase inhibitors, immune checkpoint inhibitors, or anti-VEGF therapies. Further large-scale multicenter studies are needed to confirm the advantages of vitamin K combined with TACE.

In conclusion, our study showed that combining *nontoxic* vitamin K with TACE for unresectable HCC could increase the ORR and prolong PFS when compared with TACE alone.

## Supplementary information

Supplementary figures, table and drug information

## Data Availability

The data of the study is available when requested. The corresponding author will address the requests subject to ethical consent.
