# Supplementary material for: Anticancer effects of vitamin K combined with transarterial chemoembolization in hepatocellular carcinoma, a randomized controlled trial
Source: Br J Cancer. 2025 Apr 22;132(12):1141–7. doi: 10.1038/s41416-025-03022-4 (PMC12152169; doi:10.1038/s41416-025-03022-4)
Supplement: Supplementary file 1 — Supplementary figures, table and drug information [file 41416_2025_3022_MOESM1_ESM.docx]

**Anticancer effects of vitamin K combined with transarterial chemoembolization**

**in hepatocellular carcinoma, a randomised controlled trial.**

Yoshimichi Haruna^1^, Takayuki Yakushijin^2,4^, Miho Yamakawa^3,4^, Tetsuo Nakazawa^3,4^

^1^Department of Medical Affairs, Osaka Psychiatric Medical Center, Hirakata City, Osaka Prefecture, Japan, and ^2^Department of Gastroenterology and Hepatology, ^3^Department of Diagnostic Imaging and ^4^Liver Cancer Center, Osaka General Medical Center, Osaka City, Osaka Prefecture, Japan

Contents

Fig. S1…………………………………………………………………………………………………2-3

Fig. S2………………………………………………………………………………………………….4

Fig. S3………………………………………………………………………………………………….5

Table S1………………………………………………………………………………………………...6

Drug information of vitamin K2 (menatetrenone;GLAKAY^Ⓡ^)………………………………………...7-10


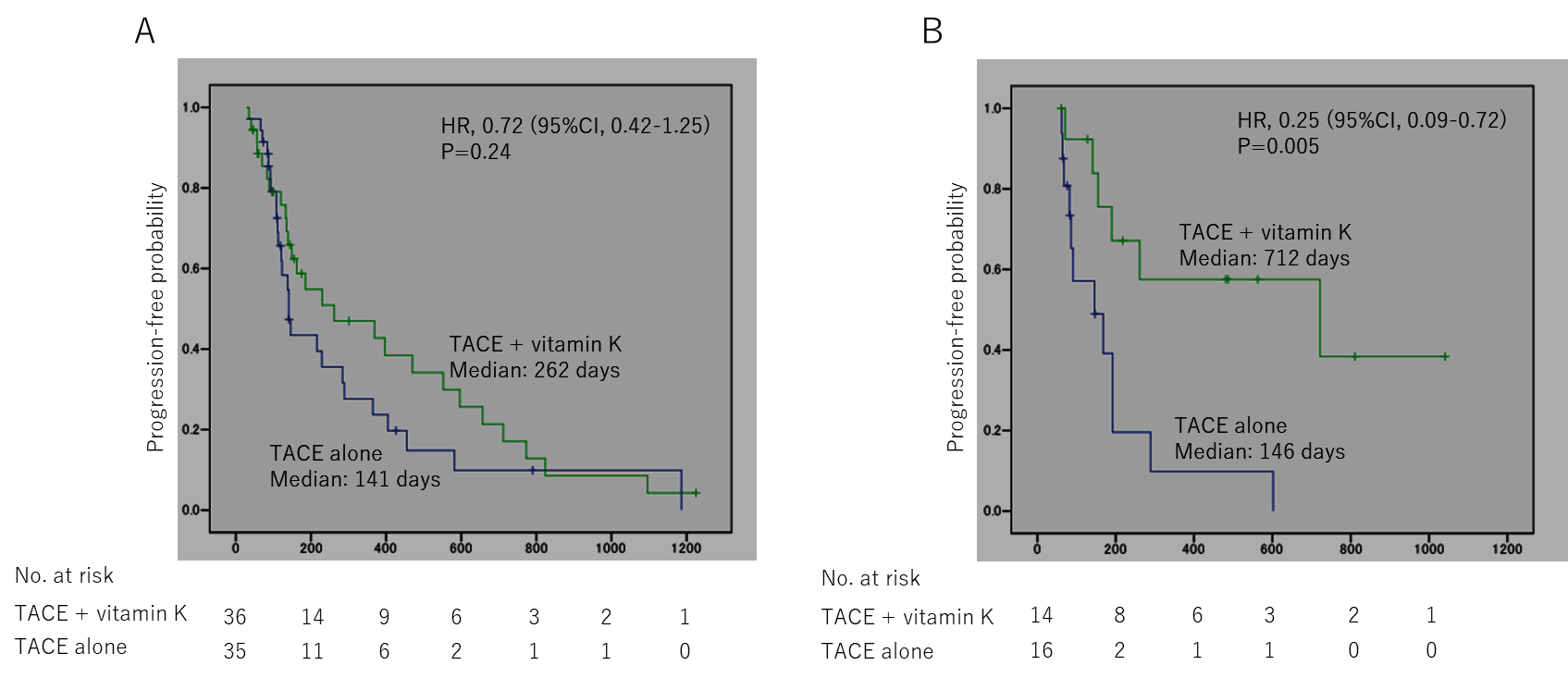


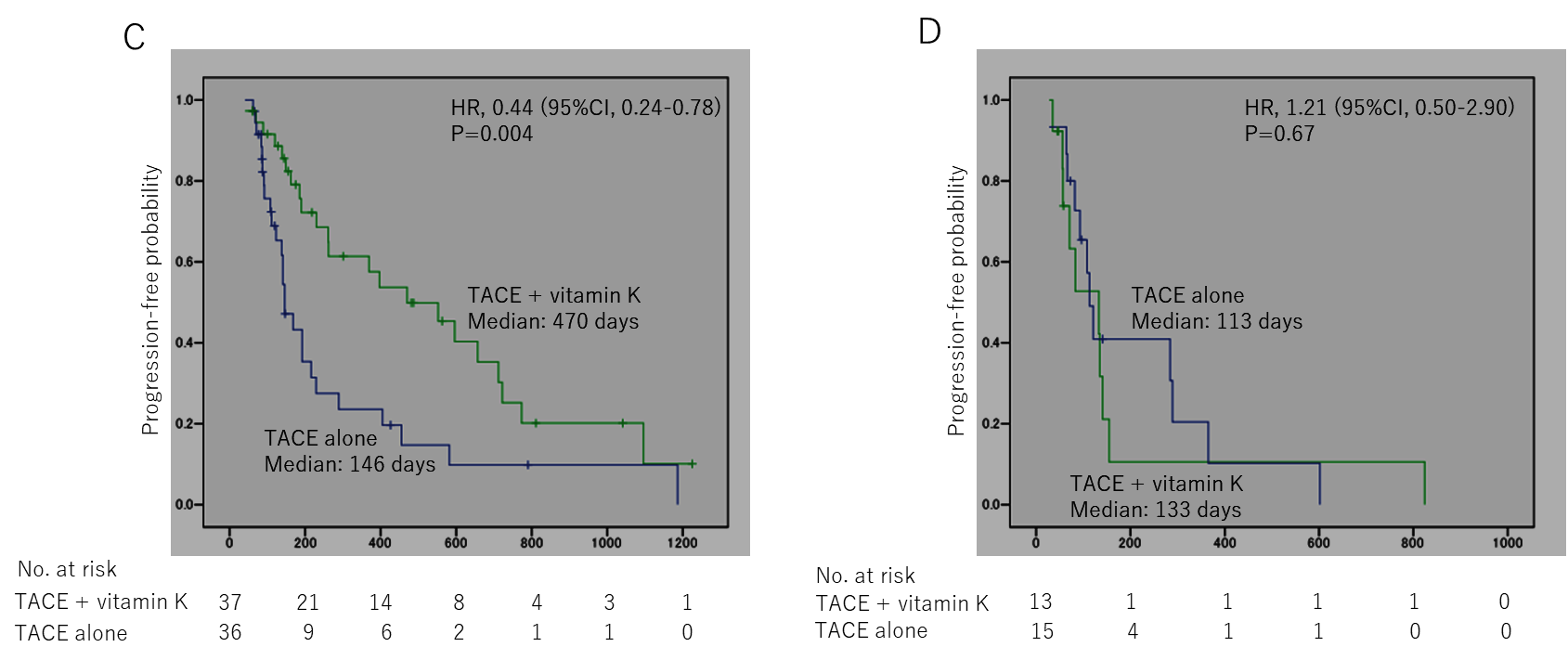

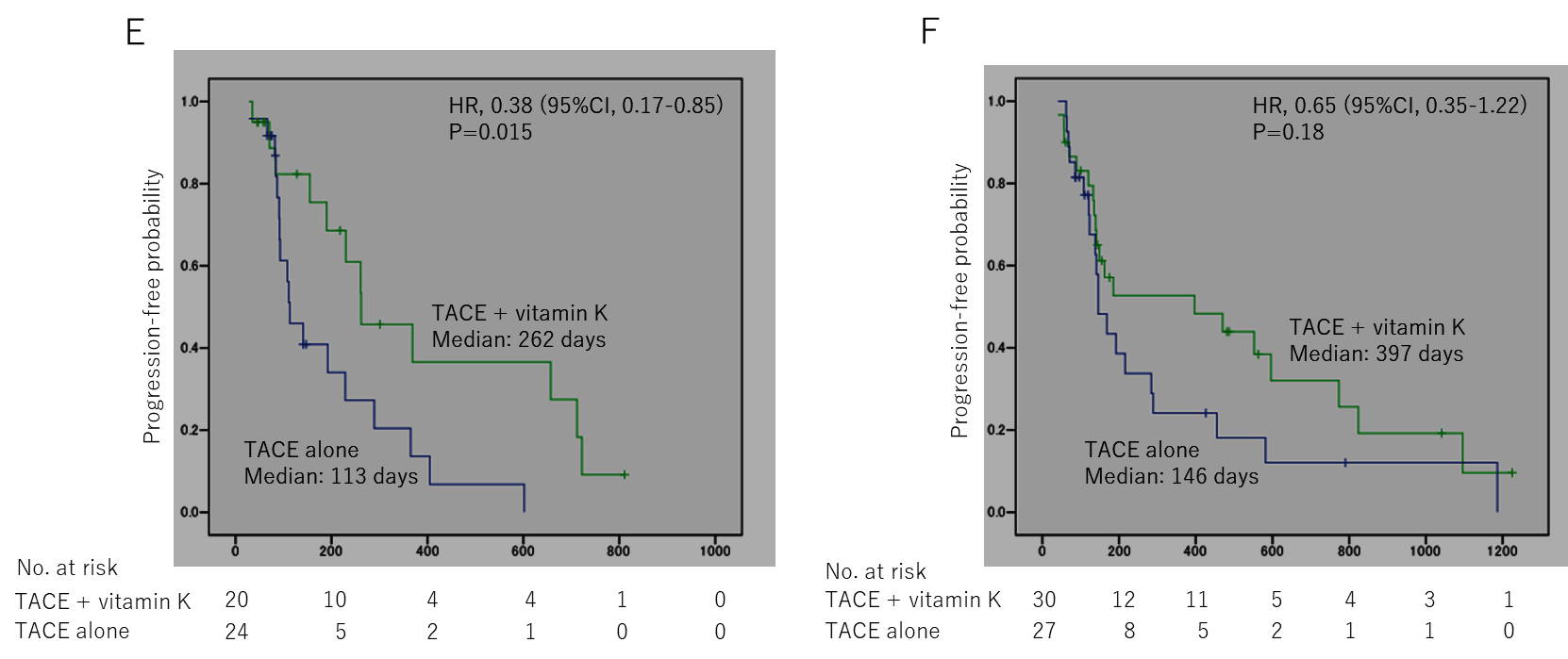


**Supplementary Fig. S1.**

**Kaplan–Meier curves of progression-free survival in male patients (A), female patients (B), patients within up-to-7 criteria (C), patients beyond up-to-7 criteria (D), patients with baseline serum DCP levels**$\geq$**100 mAU/mL (E), and patients with baseline serum DCP levels <100 mAU/mL (F)**

HR, hazard ratio; CI, confidence interval; TACE, transarterial chemoembolization; DCP, des-γ-carboxy prothrombin.


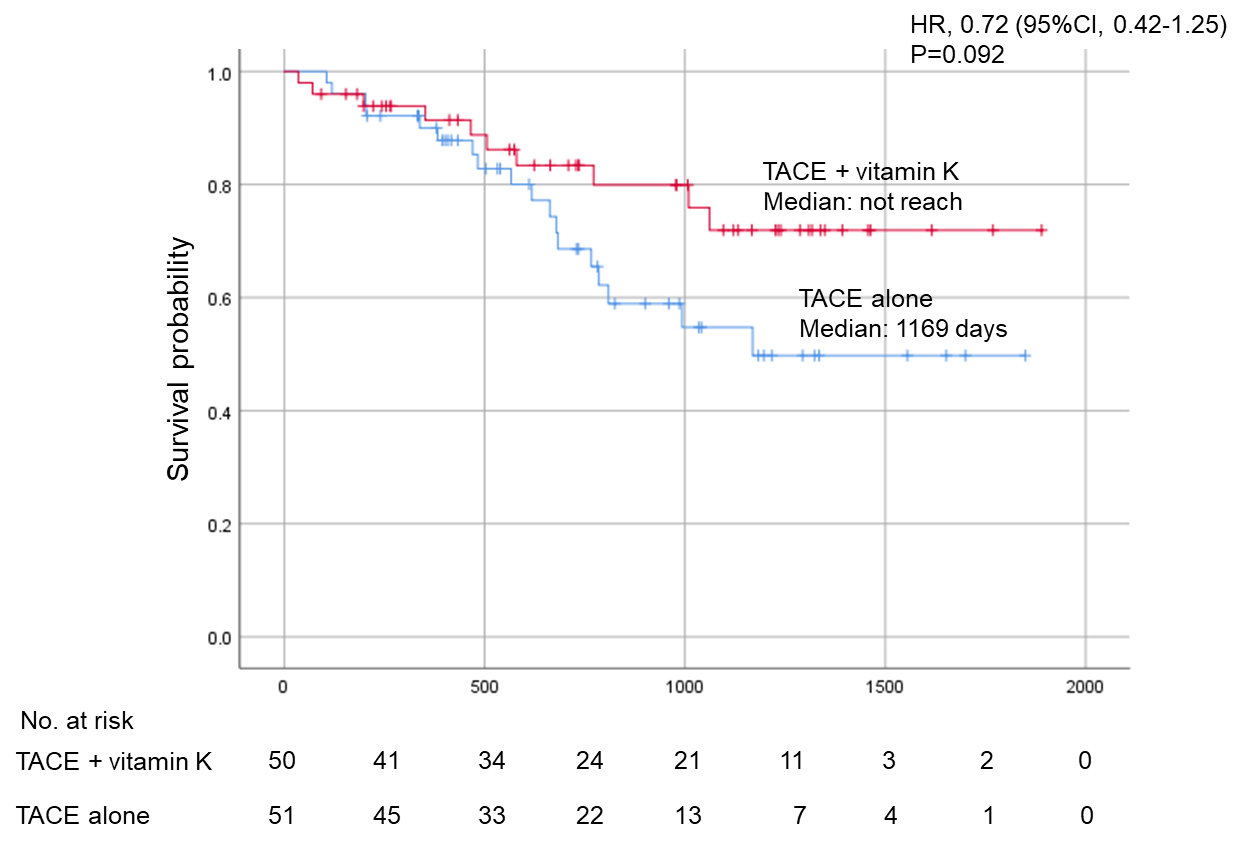


**Supplementary Fig. S2.**

**Kaplan–Meier curves of overall survival**

HR, hazard ratio; CI, confidence interval; TACE, transarterial chemoembolization.


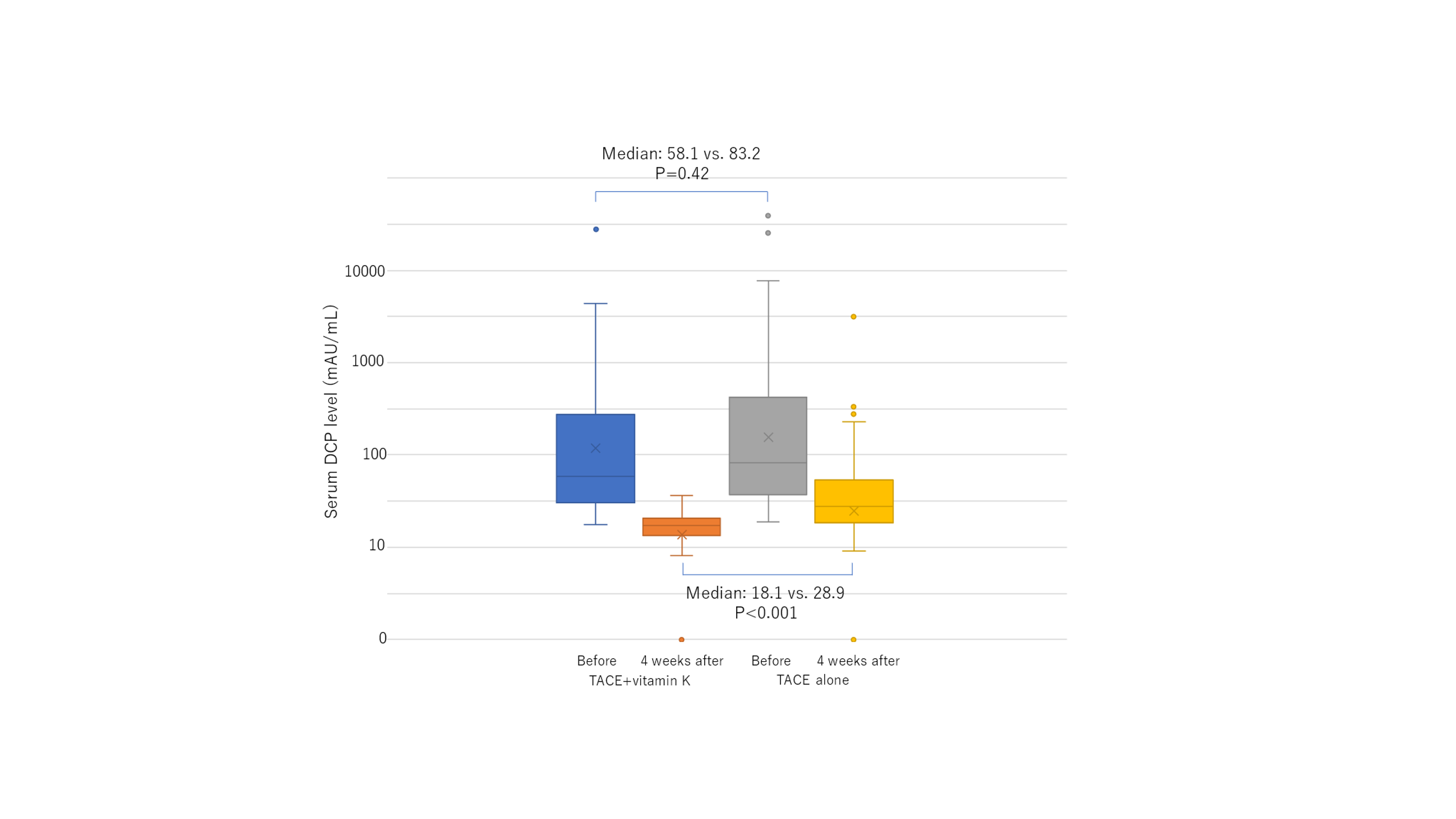


**Supplementary Fig. S3. Serum DCP levels before and four weeks after** **the treatment**

No significant differences at baseline are observed between the TACE + vitamin K and TACE alone groups (*p*=0.42); median (range) mAU/mL, 58.1 (17.7–28093.0) *vs.* 83.2 (19.0–38999.2) (Mann–Whitney U test). Four weeks post-TACE, a significant difference was observed between the two groups (*p*<0.001); 18.1 (8.1–36.4) *vs*. 28.9 (9.0–3170.7), respectively. Patients in the vitamin K dosing group show serum DCP levels below the normal upper limit (<40 mAU/mL), despite including those with PR and SD. DCP, des-γ-carboxy prothrombin; PR, partial response; SD, stable disease; TACE, transarterial chemoembolization.

**Supplementary Table S1.** **Best responses, ORR, and DCR for patients treated with TACE + vitamin K or TACE alone shown as n (%)**

|  | TACE + vitamin K (n=50) | TACE alone (n=51) | *p-*value |
| --- | --- | --- | --- |
| Best response |  |  |  |
| CR | 31 (62.0) | 24 (47.1) |  |
| PR | 17 (34.0) | 18 (35.3) |  |
| SD | 2 (4.0) | 5 (9.8) |  |
| PD | 0 (0.0) | 4 (7.8) |  |
|  |  |  | 0.052 |
| ORR | 48 (96.0) | 42 (82.4) | 0.028 |
| DCR | 50 (100.0) | 47 (92.2) | 0.061 |

CR, complete response; DCR, disease control rate; ORR, objective response rate; PD, progressive disease; PR, partial response; SD, stable disease; TACE, transarterial chemoembolization.
